# Molecular Epidemiology of *Klebsiella pneumoniae* Based on Whole-Genome Sequencing Among Hospitalized Patients in Huzhou, China: A 6-Year Surveillance Study, 2020–2025

**DOI:** 10.3390/microorganisms14071498

**Published:** 2026-07-09

**Authors:** Lei Ji, Peng Zhang, Yunfeng Zha, Fenfen Dong, Wei Yan

**Affiliations:** Microbiology Laboratory, Huzhou Center for Disease Control and Prevention (Huzhou Municipal Health Inspection and Supervision Agency), Huzhou 313000, China

**Keywords:** *Klebsiella pneumoniae*, antimicrobial resistance, virulence, whole-genome sequencing, *mcr-1*, *bla*_KPC-2_

## Abstract

*Klebsiella pneumoniae* has become a critical global public health threat due to the rapid spread of hypervirulent and multidrug-resistant clones. A total of 205 non-duplicate *K. pneumoniae* isolates were collected from seven hospitals in Huzhou, China, between January 2020 and December 2025. Antimicrobial susceptibility testing, the string test, whole-genome sequencing, and bioinformatics analysis were applied to determine resistance phenotypes, virulence features, multilocus sequence types (STs), capsular types, plasmid replicons, and phylogenetic relationships. Of the 205 isolates, 180 (87.80%) were identified as hypervirulent *K. pneumoniae* (hvKP), with ST23-K1 and ST86-K2 being the dominant clones. This unusually high hvKP detection rate may be attributable to our study population composition, with patients aged ≥ 60 years accounting for 77.08% of all subjects. High resistance rates were observed for ampicillin (73.66%), tetracycline (27.32%), and trimethoprim–sulfamethoxazole (25.85%). Eleven isolates carried *bla*_KPC-2_ and one harbored *mcr-1*, displaying corresponding drug-resistant phenotypes. The isolates exhibited high genetic diversity with 62 STs, 25 capsular types, and 38 plasmid replicons. Co-occurrence of virulence and resistance determinants was commonly detected. Five genetic lineages (A to E) were classified among all isolates, and no epidemiological outbreak clusters were detected in our study. The hvKP is highly endemic in Huzhou, while local *K. pneumoniae* isolates show high resistance to conventional antibiotics but remain largely susceptible to last-resort antimicrobials. The coexistence of hypervirulence and antimicrobial resistance underscores the urgent need for continuous genomic surveillance to curb the dissemination of high-risk clones.

## 1. Introduction

*Klebsiella pneumoniae* represents one of the most prevalent opportunistic pathogens in clinical settings [1]. As a major etiological agent of hospital-acquired infections, it is frequently implicated in a wide spectrum of infectious diseases, including pneumonia, urinary tract infections, and bloodstream infections. Notably, infections caused by *K. pneumoniae* are particularly common among immunocompromised individuals, such as neonates and the elderly [2]. Outside healthcare settings, *K. pneumoniae* is also recognized as a causative pathogen responsible for a range of community-acquired infections, including pyogenic liver abscess, endophthalmitis, and meningitis [3]. Whole-genome sequencing (WGS) of *K. pneumoniae* reveals a pathogen in continuous evolutionary flux, acquiring resistance and virulence determinants through both clonal expansion and horizontal gene transfer across diverse ecological niches. Whole-genome sequencing has transformed our understanding of *K. pneumoniae* epidemiology by enabling the characterization of sequence types, capsular loci, plasmid replicons, and resistance gene environments, providing resolution far superior to conventional phenotypic surveillance. This genomic framework is now considered indispensable for tracking the emergence of high-risk clones in healthcare settings and aligns with previous studies that have highlighted the importance of understanding the genetic diversity, ecological adaptation, and pathogenic potential of *K. pneumoniae* in both clinical and environmental contexts [4,5,6]. Currently, *K. pneumoniae* has evolved multiple distinct strains exhibiting diverse resistance and virulence phenotypes. These strains include hypervirulent *K. pneumoniae* (hvKP), carbapenem-resistant *K. pneumoniae* (CRKP), and colistin-resistant *K. pneumoniae* (CoRKp), thereby posing a substantial threat to human health [7].

First identified in patients with liver abscess in Taiwan in 1986 [8], hvKP can lead to life-threatening infections. It frequently affects relatively young and healthy patients, who present with multisite infections or develop subsequent metastatic spread [9]. In China, CRKP accounts for approximately 90% of clinical carbapenem-resistant *Enterobacteriaceae* (CRE) infections [10], a high prevalence driven by widespread antimicrobial use that confers a strong selective advantage. With the increasing prevalence of CRKP, colistin has re-emerged as “the last option” against CRKP infections. However, the rising reports of CoRKp pose a major challenge to healthcare [11,12]. Moreover, the acquisition of extensive or pan-antimicrobial resistance may lead to the emergence of untreatable “superbugs”, and the co-occurrence of multidrug resistance and hypervirulence further severely limits available therapeutic options [9,13]. In recent years, China has been identified as a major endemic region for carbapenem-resistant hypervirulent *K. pneumoniae* (CR-hvKP), with the highest number of reported cases [14]. The prevalence of CR-hvKP has shown an increasing trend in more than half of China’s regions, with significant regional disparities. The highest prevalence rates were observed in Zhejiang (28.5%), Jiangsu (19.4%), and Beijing (9.7%) [9].

*K. pneumoniae* exhibits marked regional heterogeneity in prevalence, drug resistance, virulence, and capsular genotypes [15,16]. However, little is known about the molecular epidemiology of *K. pneumoniae* and CR-hvKP in Huzhou, a city in Zhejiang Province, China. In this article, we analyzed the epidemic characteristics, capsular types, and STs of *K. pneumoniae*. We also performed antimicrobial susceptibility testing (AST) and WGS to identify antibiotic resistance genes (ARGs) and virulence factors (VFs) and conducted single nucleotide polymorphism (SNP) analysis to determine phylogenetic relationships.

## 2. Materials and Methods

### 2.1. Bacterial Isolates and Identification

A total of 1947 clinical samples were collected from patients with *K. pneumoniae* infection who were admitted to 7 hospitals across 5 counties of Huzhou between 2020 and 2025, of which 205 non-duplicate isolates (one per patient, 10.53%) were confirmed as *K. pneumoniae* and included in the final analysis. The duplicate isolates were excluded by matching patient identity information including full names, and unified inclusion criteria were implemented in all participating hospitals with only inpatient specimens enrolled and outpatient samples excluded. The strains were mainly isolated from sputum, urine, and blood samples of the patients. All samples were inoculated onto Columbia blood agar plates (Chromogar, La Plaine Saint-Denis, France) and incubated at 36 °C for 18 h. After incubation, suspected colonies were selected and subsequently identified using matrix-assisted laser desorption/ionization time-of-flight mass spectrometry (MALDI-TOF MS) (bioMérieux, Craponne, France). All the isolates were stored in 25% glycerol broth in a freezer at −80 °C.

### 2.2. Antimicrobial Susceptibility Testing

Antimicrobial susceptibility testing was performed using the broth microdilution method. Phenotypic resistance was interpreted according to the breakpoints established by the CLSI (Clinical and Laboratory Standards Institute) and EUCAST, using minimum inhibitory concentration (MIC) values [17,18]. The 17 antimicrobials (Thermo, Waltham, MA, USA) including ampicillin/sulbactam (AMS), ampicillin (AMP), streptomycin (STR), amikacin (AMI), azithromycin (AZM), nalidixic (NAL), ciprofloxacin (CIP), tigecycline (TIG), tetracycline (TET), ceftazidime/avibactam (CZA), ceftazidime (CAZ), cefotaxime (CTX), meropenem (MEM), ertapenem (ETP), colistin (CT), trimethoprim-sulfamethoxazole (SXT) and chloramphenicol (CHL) were tested in this assay. *Escherichia coli* ATCC 25922 was used for quality control.

### 2.3. String Test

The string test was used to detect the hypermucoviscous phenotype. Strains were cultured overnight at 37 °C on 5% sheep blood Columbia agar. A single colony was gently stretched with an inoculation loop; a string length over 5 mm was defined as a positive hypermucoviscous phenotype [19]. The string test served merely as a supplementary backup assay; the primary classification of hvKP was determined based on virulence genes.

### 2.4. Whole Genome Sequencing

The genomic DNA was extracted using a QIAamp DNA Mini Kit (Qiagen, Hilden, Germany) from overnight cultures. Whole-genome sequencing was performed on Illumina NextSeq 550 (Illumina Inc., San Diego, CA, USA) with a 150-bp paired-end library. The raw data were quality-controlled using FastQC v0.11.5 and Trimmomatic v0.36 [20]; genome assembly was conducted with SPAdes v3.13.0 [21], assembly quality was assessed by CheckM, and only genomic data with no more than 100 scaffolds, genome coverage of at least 95% and a Q30 quality score no lower than 85% were retained for subsequent analysis.

### 2.5. Bioinformatics Analysis

Multilocus sequence typing (MLST), capsular typing and virulence scoring were conducted via Kleborate v3.2.4 (http://github.com/katholt/Kleborate (accessed on 13 February 2026)) [22]. This novel tool assesses hvKP virulence and assigns a corresponding score ranging from 0 to 5. The minimum spanning tree was generated using BioNumerics software v7.6. The PlasmidFinder v2.1 (https://cge.food.dtu.dk/services/PlasmidFinder/ (accessed on 11 February 2026)) with a similarity cut-off of 90% was used to determine the different plasmid replicons. ARGs were predicted using the CARD database (v3.2.7) [23], while DIAMOND v0.9.19 was employed to analyze VFs against the VFDB database (v20250227) [24]. The similarity cut-off and coverage threshold were both set at 90–100%. The isolates carrying any one of the four genes *rmpA*, *iucA*, *iroB*, or *rmpA2* were defined as hvKP in this study.

Using the HS11286 reference sequence, SNP calling was performed on genomic data from the studied isolates using Snippy v4.4.5. HS11286 is a manually curated, high-quality, and complete reference genome available in the National Center for Biotechnology Information (NCBI) Genome database. The resulting outputs were combined via the snippy-multi pipeline to generate a multi-sample SNP matrix. Subsequently, Gubbinsv3.0.0 [25] was applied to identify and eliminate recombinant regions from the concatenated SNP dataset. Finally, a maximum-likelihood phylogeny based on the core SNPs was constructed using IQ-TREE v2.1.11 [26]. The lineages were defined by visual inspection of the phylogenetic tree. Genomic maps of *mcr-1*-carrying isolates were constructed with Proksee v1.0.0 (https://proksee.ca/ (accessed on 9 February 2026)). Graphical visualization of bar plots, heatmaps, and phylogenetic trees was performed using ChiPlot [27]. In addition, the reference sequences of *mcr-1*-positive *K. pneumoniae* were retrieved from the NCBI Pathogen Detection database (https://www.ncbi.nlm.nih.gov/pathogens/ (accessed on 14 January 2026)) using the search query: AMR_genotypes: *mcr-1* ANDtaxgroup_name: “*Klebsiella pneumoniae*”.

### 2.6. Statistical Analysis

Data were analyzed using SPSS 23.0 (IBM, New York, NY, USA). Chi-square tests were applied to compare Salmonella infection rates by sex and age, and *p* < 0.05 was considered statistically significant.

## 3. Results

### 3.1. Prevalence of K. pneumoniae Among Patients

**Figure 1:**
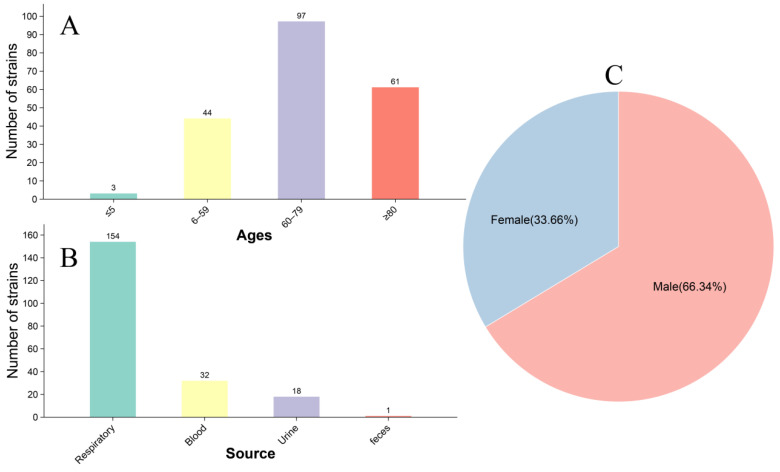
Epidemiological information of the 205 *K. pneumoniae* isolates. (**A**) The number of *K. pneumoniae* isolates in each age group; (**B**) the number of K. pneumoniae isolates from different clinical sources; (**C**) sex composition ratios of *K. pneumoniae* isolates.

In this study, a total of 205 strains were isolated, including 180 hvKP, with 11 *bla*_KPC-2_-positive *K. pneumoniae* and one *mcr-1*-positive *K. pneumoniae.* An overview of the genome sequences is provided in Supplementary Table S1. The age of patients ranged from 1 to 97 years, with ≥80 years accounting for 29.76%, 60–79 years accounting for 47.32%, 6–59 years accounting for 21.46% and ≤5 years accounting for 1.46% (Figure 1A). The proportion of isolates from the respiratory tract, blood, urine, and feces were 75.12%, 15.61%, 8.78%, and 0.49%, respectively (Figure 1B). Notably, *mcr-1*-positive *K. pneumoniae* was isolated from fecal specimens. Of the 205 bacterial strains, 136 were isolated from male patients (Figure 1C). A Chi-square test for independence was performed to examine the association between sex and age groups. No statistically significant association was observed between sex and age distribution (χ^2^ = 3.995, *p* = 0.262), indicating that the male-to-female ratio was comparable across the four age groups.

### 3.2. Antimicrobial Susceptibility of Isolated Strains

As shown in Table 1, the 205 isolates exhibited a high level of resistance to AMP (73.66%, 151/205), TET (27.32%, 56/205), and SXT (25.85%, 53/205). A minority of isolates were resistant to CZA (1.46%, 3/205), CT (2.93%, 6/205), and TIG (8.29%, 17/205). Additionally, each of the 11 *bla*_KPC-2_-positive *K. Pneumoniae* was resistant to ETP and MEM, and the *mcr-1*-positive *K. pneumoniae* was resistant to CT (Supplementary Table S2). Among all 205 isolates, the overall MDR rate was 18.54% (38/205), with the most prevalent MDR profile being ETP-MEM-CTX-CAZ-TET-CIP-AMI-AMP-AMS, accounting for 34.21% (13/38) of the MDR isolates.

### 3.3. Distribution of Capsule Types and Plasmids

**Figure 2:**
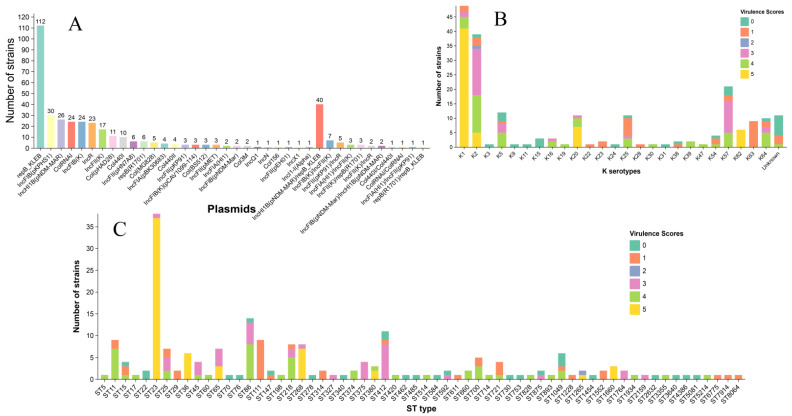
Distribution of plasmid replicons, K-serotypes and ST genotypes of 205 clinical *K. pneumoniae* isolates. (**A**) Prevalence of various plasmid replicon types; (**B**) distribution of capsule types; (**C**) MLST-based population structure of isolates.

A total of 38 plasmid types were identified, with only four strains harboring no detectable plasmids (Figure 2A). Of all the plasmids identified, repB_KLEB (54.63%, 112/205) exhibited the highest prevalence, followed by IncHI1B (pNDM-MAR)/repB_KLEB (19.51%, 40/205) and IncFIB (pKPHS1) (14.63%, 30/205). The *bla*_KPC-2_-positive and *mcr-1*-positive *K. pneumoniae* isolates generally harbored a large number of plasmids, ranging from 2 to 6. Based on whole-genome sequencing and plasmid replicon typing, the *bla*_KPC-2_ and *mcr-1* genes were confirmed to be plasmid-borne. The capsular diversity is typically associated with pathogenicity, and high virulence scores (≥3) were predominantly concentrated in K1 and K2 (Figure 2B). Among the capsular types, K1 exhibited the highest detection rate (24.39%, 50/205), followed by K2 (18.54%, 38/205) and K57 (10.24%, 21/205), although prediction failed for 11 strains. K64 was the predominant capsule type among *bla*_KPC-2_-positive isolates, accounting for 54.55% (6/11).

### 3.4. K. pneumoniae MLST

**Figure 3:**
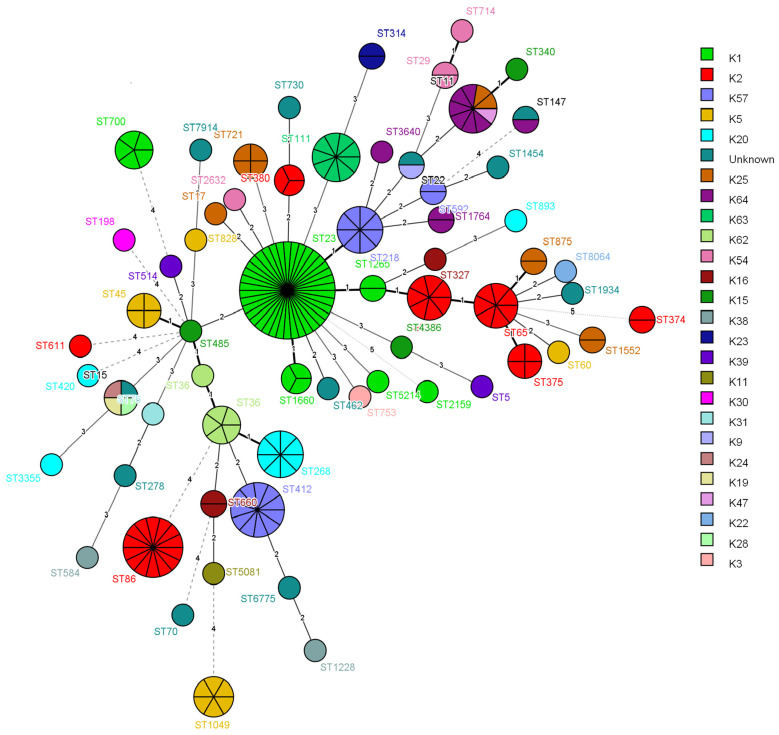
The minimum spanning tree of 205 *K. pneumoniae* isolates. The numbers on the branches represent the number of differences in the seven housekeeping genes used to determine STs.

A total of 62 distinct STs were identified, among which three accounted for over 5% of all strains: ST23 (18.54%), ST86 (6.83%), and ST412 (5.36%). Overall, these three STs accounted for 30.73% of the total population (Figure 2C). Virulence scores showed a strong correlation with STs. In total, 97.37% of ST23 isolates had a maximum virulence score of 5. Nine of the 11 *bla*_KPC-2_-positive *K. Pneumoniae* isolates belonged to ST11, while the *mcr*-1-positive isolate belonged to ST753. The minimum spanning tree based on seven housekeeping genes is shown in Figure 3.

### 3.5. Phylogeny Analysis

**Figure 4:**
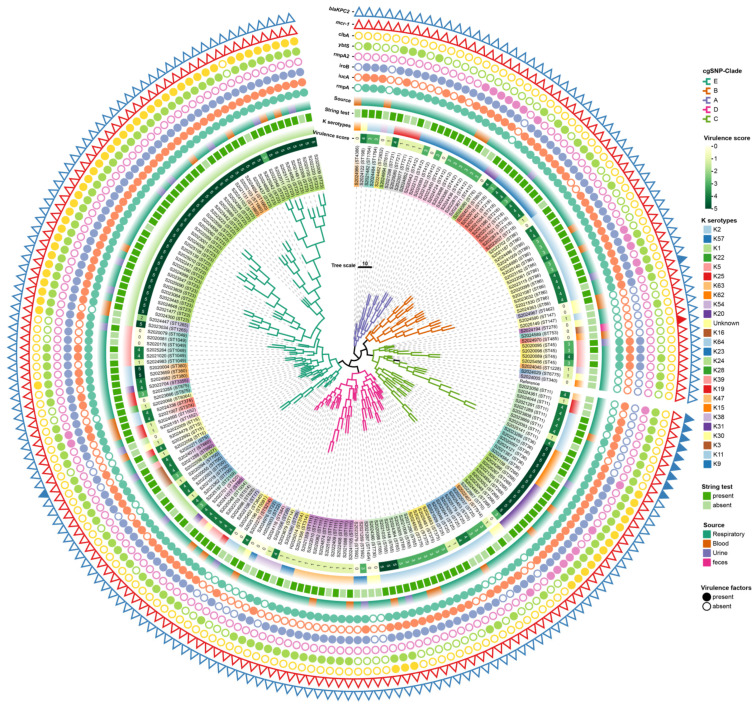
The core genome SNP-based phylogenomic tree of 205 *K. pneumoniae* isolates obtained from clinical samples in Huzhou. The STs, virulence scores, capsule types, string test, source, virulence genes, resistance genes, and lineages have been represented.

The 205 isolates displayed high genetic diversity, and phylogenetic reconstruction classified them into five lineages (A–E) (Figure 4). Lineage A comprised 21 isolates, with the predominant ST and capsule type being ST412 and K54, respectively (52.38%, 11/21). A total of 24 strains belonged to lineage B, with ST86 and K2 being the predominant ST and capsule type, respectively (58.33%, 14/24). Virulence scores of strains belonging to lineages A and B were generally low, with no strain scoring 5. Lineage C comprised 36 isolates with diverse ST and capsule type distribution and no dominant type. Notably, nine *bla*_KPC-2_-positive and 1 *mcr-1*-positive *K. pneumoniae* isolates belonged to this lineage. Lineage D exhibited no dominant STs or capsule types, with ST111 (20.00%, 9/45) and K2 (40.00%, 18/45) being the most prevalent. Lineage E contained the largest number of isolates, with a total of 79 strains. The predominant STs and capsule types were ST23 (48.10%, 38/79) and K1 (62.03%, 49/79), respectively. Meanwhile, Lineage E represented the main cluster of hvKP. Only three isolates in this lineage lacked any of the four major virulence genes, namely *rmpA*, *iucA*, *iroB*, and *rmpA2*. Furthermore, 72.88% (43/59) of strains with a virulence score of 5 were distributed in Lineage E.

### 3.6. Virulence Analysis of K. pneumoniae

A total of 150 *K. pneumoniae* strains (73.17%) were identified as hypermucoviscous by the string test. Among these, 112 strains were isolated from the respiratory tract, 25 from the blood and 13 from urine.

A total of 301 virulence factors (VFs) were identified in *K. pneumoniae* (Supplementary Table S3), including adherence (type 1 fimbriae), biofilm formation (type 3 fimbriae), efflux pumps such as AcrAB, immune evasion (capsule), iron uptake (enterobactin, salmochelin, and yersiniabactin), regulatory (RcsAB), and secretion system (T6SS). We then annotated the hypervirulence-associated genes (*rmpA*, *iucA*, *iroB*, *rmpA2*, *ybtS* and *clbA*) in Figure 4. The positive rates of these six genes were 82.44%, 69.27%, 82.44%, 11.22%, 67.32%, and 28.78%, respectively. A total of 180 isolates harboring at least one of the four genes (r*mpA*, *iucA*, *iroB*, and *rmpA2*) were identified as hvKP. Among the 205 isolates, nine (4.39%) were *bla*_KPC-2_-positive hvKP “superbugs” (Supplementary Table S4). K64 was the predominant K serotype (6/9, 66.67%), and *iucA* was present in all these nine isolates. The majority (8/9, 88.89%) belonged to ST11 and formed a single clonal cluster, while one isolate (S2021530) belonged to ST86-K2 and carried all four virulence genes. These results indicate that the ST11-K64 clone is the dominant “superbug” lineage in our study. In addition, the single *mcr*-1-positive *K. pneumoniae* isolate was characterized as non-hypervirulent.

The distribution of high virulence scores was as follows: 59 isolates exhibited a score of 5, 47 had a score of 4, and 37 had a score of 3 (Figure 4). Overall, the proportion of high virulence scores (≥3) was relatively high (69.76%). For the 59 isolates with a virulence score of 5, the dominant ST was ST23 (62.71%), the main capsule type was K1 (69.49%), and the positive rate of the string test was 89.83%. The four hypervirulence-associated genes (*rmpA*, *iucA*, *iroB*, and *rmpA2*) were detected at rates of 100.00%, 100.00%, 100.00%, and 10.17%, respectively.

### 3.7. Antibiotic Resistance Determinants

**Figure 5:**
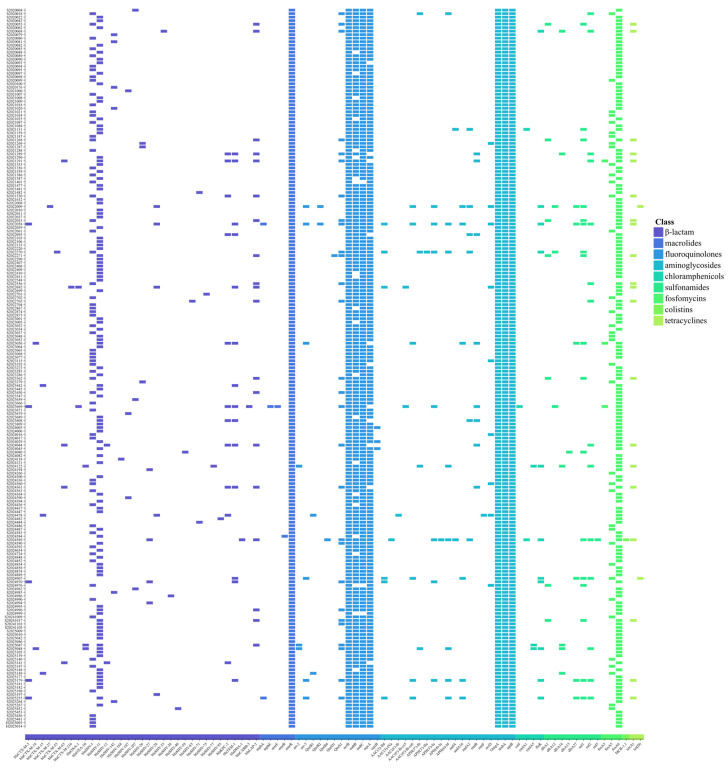
Annotation heatmap of antimicrobial resistance genes among 205 *K. pneumoniae* isolates.

According to CARD analysis, 87 ARGs were found in 205 *K. pneumoniae* isolates, including *bla*_KPC-2_, *mcr-1*, *blaCTX-M-3*, and so on (Supplementary Table S5, Figure 5). These ARGs can be classified into nine major categories, including β-lactams, macrolides, fluoroquinolones, aminoglycosides, chloramphenicols, sulfonamides, fosfomycins, colistins and tetracyclines. Among them, the detection rate of β-lactamase was also high, accounting for 98.54%. Notably, the *bla*_KPC-2_ gene was the most frequently detected carbapenemase gene (*n* = 11), whereas only one isolate carried the *mcr-1* gene.

**Figure 6:**
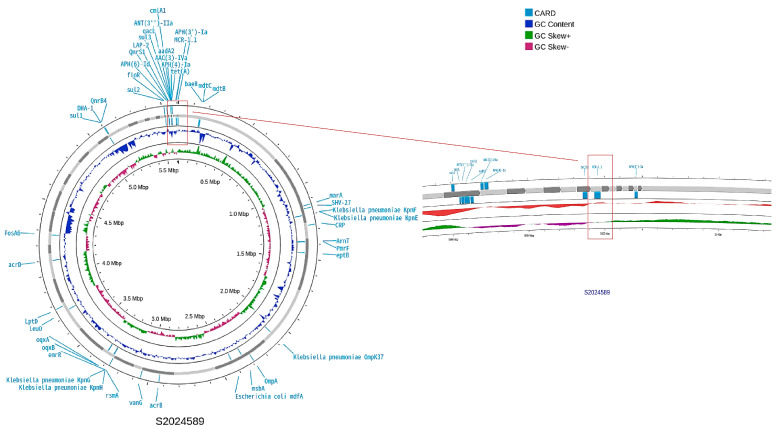
Circular genome map of the *mcr*-1-positive *K. pneumoniae* strain isolated in this study.

**Figure 7:**
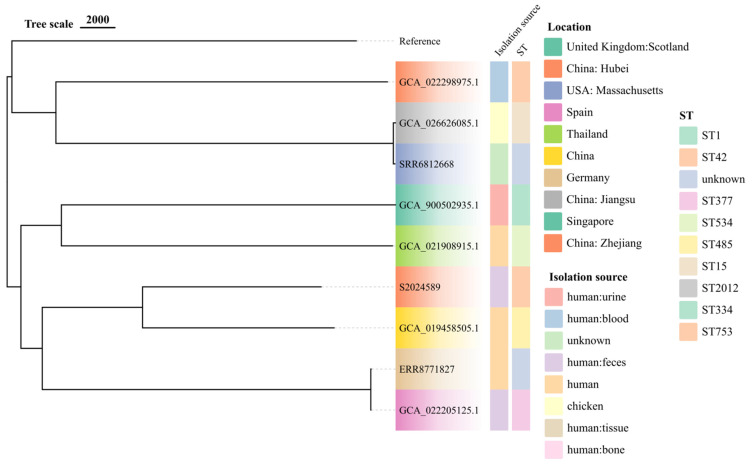
The core genome SNP-based phylogenomic tree of our *mcr*-1-positive *K. pneumoniae* genome compared to other published genomes of similar microorganisms. The *mcr-1*-harboring isolates in this study is “S2024589”.

One strain carrying the *mcr-1* gene was resistant to colistin and belonged to ST753 (Figure 6). We retrieved an additional eight genomes of global *mcr-1*-harboring *K. pneumoniae* isolates from NCBI to identify the genomic relationships (Figure 7). The SNP phylogenetic analysis classified the nine isolates into two distinct clades. Among all comparisons, the isolate S2024589 from this study exhibited the lowest SNP divergence relative to GCA_019458505.1, which still totaled 15,171 SNPs (Supplementary Table S6).

## 4. Discussion

In this study, we performed a long-term molecular epidemiological investigation of clinical *K. pneumoniae* isolates. Our results indicate the hvKP, CRKP, and CoRKp were widely distributed in Huzhou. Notably, hvKP was absolutely predominant (87.8%), which is higher than the national averages reported by CHINET/CARSS. This likely reflects two factors. First, 77.08% of our patients were aged ≥60 years, and advanced age is a known risk factor for hvKP infection. Previous study has documented that elderly individuals are susceptible to *K. pneumoniae* infections, mainly due to the presence of at least one underlying disease and impaired immune function [28]. Second, our genotypic definition (any one of *rmpA*, *rmpA2*, *iucA*, or *iroB*) is broader than some surveillance definitions that rely on phenotypic tests or stricter gene combinations. The hvKP infections tend to cause multifocal and atypical clinical manifestations, often accompanied by bacteremia and distant metastatic infections [2,29].

The increasing prevalence of antimicrobial resistance, especially carbapenem and colistin resistance in *K. pneumoniae*, has posed a worldwide threat for many decades [11,30]. This may be attributed to the misuse of antibiotics in clinical, environmental, and agricultural settings [31,32]. Various mechanisms contribute to antimicrobial resistance in *K. pneumoniae*, including the acquisition and transfer of resistance gene-bearing plasmids and chromosomal mutations [33]. The high resistance rates observed for AMP (73.66%), TET (27.32%), and SXT (25.85%) in this study are largely attributable to the presence of their cognate resistance genes, most notably *bla*_SHVX_, *sulX*, and *tet(A).* These findings are also consistent with our previous study [34]. Notably, the resistance rates to carbapenems are not low, as the resistance rates for ETP and MEM are 13.66% and 11.71% respectively, underscoring the potential threats to public health. However, colistin, a last-resort agent for Gram-negative infections, still retained favorable antimicrobial activity, with a resistance rate of only 2.93% [35]. Interestingly, 98.54% of the isolates exhibited susceptibility to CZA, suggesting that CZA may serve as a potential therapeutic option for *K. pneumoniae* infections [36,37].

Plasmids are important vehicles for the transfer of antimicrobial resistance and virulence between bacterial cells [2]. In this study, repB_KLEB was the most prevalent, representing a key type of hypervirulent plasmid in *K. pneumoniae*. It can encode and disseminate hypervirulent phenotypes, endowing bacterial strains with enhanced invasiveness and a hypermucoviscous phenotype [38]. Large conjugative (self-transmissible) plasmids, including IncR and IncFII, can mediate the horizontal acquisition of antimicrobial resistance genes in *K. pneumoniae* [39].

Capsule type analysis offered insights into genetic diversity and relatedness. A total of 25 capsular types were identified in this study. We demonstrated that K1, K2 and K57 were the dominant capsular types in Huzhou, collectively accounting for 53.17% of all isolates. To date, more than 70 capsule types have been identified using traditional serological typing methods [40]. In murine infection models, K1 and K2 have been linked to invasive infections and elevated pathogenicity [41]. These two capsule types are also highly conserved among hypervirulent clonal lineages, with K1 predominantly found in ST23 and K2 in other clonal groups [42]. In this study, all 38 ST23 isolates exhibited the K1 capsular type. Among the remaining 12 K1 isolates, ST700 was the predominant ST. In contrast, the 38 K2 isolates were distributed across seven distinct sequence types, displaying a relatively scattered distribution pattern, which is consistent with a previous report [43]. Most hypervirulent infections are caused by a limited number of dominant clones (K1 and K2), which usually harbor a combination of virulence-related allelic variants of core pathogenicity determinants [44]. However, strains with non-K1 or non-K2 (e.g., K57) have also been reported to cause hypervirulent infections [45].

Many studies have attributed the majority of CRKP infections to a limited number of STs, such as ST11 and ST15 [7,46]. Our results also corroborate this observation, as ST11 accounted for 81.82% of all CRKP isolates. Similarly, hvKP infections were predominantly caused by a single clonal lineage, with ST23 being the most common type [47]. Hypervirulent strains are highly prevalent in the Asia–Pacific region, and CG23 represents one of the most common clones associated with *K. pneumoniae* bloodstream infections in this region [48]. Minimum spanning tree analysis further demonstrated that all STs were centered on ST23 and radiated outward. Notably, the CoRKp strain identified in this study belonged to ST753, which differs from earlier reports in China [11].

The distribution of SNP also reflected diversity among *K. pneumoniae* isolates from patients, which is consistent with the previous studies [34]. This genetic diversity may be attributed to the horizontal transfer of mobile genetic elements (MGEs), such as plasmids and transposons, as well as the selective pressure exerted by clinical antibiotic use and host microenvironment [49]. Strains belonging to the same STs exhibited closer genetic relatedness and tended to harbor consistent virulence and antimicrobial resistance genes. The clear differentiation of virulence and drug resistance profiles among different lineages—with lineages A and B being low-virulence, lineages C and D carrying multidrug-resistant (MDR)-related genes, and lineage E being the main hvKP cluster—highlights the need for targeted surveillance and intervention strategies.

The hypermucoviscous phenotype of *K. pneumoniae* is closely associated with severe invasive infections, which often lead to metastatic spread and poor clinical outcomes [50]. It could produce a dense, viscous capsule that confers enhanced virulence properties, including resistance to phagocytosis and the ability to form biofilms [3]. For this reason, early investigations adopted a positive string test as the diagnostic criterion for hvKP. However, growing evidence indicates that the presence of the hypermucoviscous phenotype does not necessarily reflect the expression of hypervirulence in *K. pneumoniae* [51]. Several critical virulence factors, such as *iroB*, *iucA*, *rmpA*, and *rmpA2*, can serve as highly accurate biomarkers to distinguish hvKP from classical *K. pneumoniae* (cKP) [45]. Our molecular epidemiological data showed that the majority of *K. pneumoniae* isolates harbored one or more of these four virulence genes, indicating the endemic prevalence of hypervirulent strains in Huzhou. Among these, nine *bla*_KPC-2_-positive hypervirulent isolates were identified, which are also referred to as “superbugs” and have become widely prevalent in China in recent years [9]. The *iroB* and *iucA* encode two siderophores expressed by hvKp isolates. Siderophores enable *K. pneumoniae* to compete with the host for iron acquisition, thereby facilitating bacterial survival in iron-depleted infectious microenvironments [1]. In contrast, *rmpA* and *rmpA2* mediate the synthesis of capsular polysaccharides, leading to hypermucoviscosity in bacterial colonies [45]. To facilitate host colonization, clinical strains of *K. pneumoniae* commonly express type 1 and type 3 fimbriae, which enable their adherence to host cells [52]. Elevated expression of AcrAB is not only pivotal in mediating resistance to fluoroquinolones and β-lactams, but also provides a virulence-related advantage by improving tolerance to antimicrobial peptides secreted in the lung during innate immune defense [53]. Beyond classical resistance gene acquisition, biofilm formation by *K. pneumoniae* is a critical virulence determinant that amplifies antimicrobial tolerance and complicates eradication in clinical settings. The co-expression of type 1 and type 3 fimbriae with hypermucoviscosity further promotes surface adherence and persistent infection. Disrupting biofilm formation has therefore emerged as a promising adjunct therapeutic strategy and holds potential value in controlling antibiotic-resistant hvKP [9,54,55]. RcsAB, as a transcriptional regulator governing exopolysaccharide biosynthesis, together with the T6SS that mediates the disruption of protein targets, collectively contributes to the pathogenicity of *K. pneumoniae* isolates [56]. Virulence scoring is performed based on the clinical risk linked to the identified virulence loci, depending on the presence of key loci associated with elevated risk [22]. Nevertheless, it should be emphasized that these scores only reflect the presence of specific genetic determinants and do not directly predict clinical virulence.

The *bla*_KPC-2_ gene is dominant in clinical settings across China, as it can efficiently hydrolyze the majority of β-lactam antibiotics as well as β-lactamase inhibitors [57]. It can be disseminated via clonal expansion, horizontal gene transfer, and plasmid-mediated transmission [33]. Additionally, isolates carrying *bla*_KPC-2_ exhibit a tendency to obtain additional virulence genes and spread persistently, which warrants considerable attention [58]. Studies from Southwest China and Huaian, China, have also reported the predominance of ST11-K64 CRKP, with nearly 100% blaKPC-2 carriage [59,60]. The detection of *bla*_KPC-2_ in 11 isolates underscores the urgent need for evidence-based infection control measures at the institutional level. Hospital-acquired carbapenem-resistant Enterobacteriaceae are amenable to containment through active surveillance, structured audit programs, and prompt physician notification following positive culture results—approaches that have been shown to significantly reduce acquisition rates in tertiary care settings. The implementation of such surveillance bundles, alongside routine WGS, should be considered integral to national antimicrobial stewardship frameworks [61,62]. As therapeutic choices for carbapenem-resistant strains are scarce, colistin has once again become the last-resort antibiotic for the treatment of these infections [63]. Following the emergence of the *mcr-1* in 2015, research attention has largely focused on plasmid-mediated resistance, owing to its high capacity for rapid horizontal gene transfer [64]. Phylogenetic analysis revealed that it was distantly related to other genomes, and its origin remains to be further investigated. A number of isolates carried resistance genes while remaining phenotypically susceptible to antibiotics. This might result from insufficient gene expression or low-level resistance that did not reach the diagnostic breakpoint [65]. Similarly, phenotypic carbapenem resistance without corresponding resistance genes detected may result from mutations or transcriptional silencing of outer membrane porin genes (such as ompK35 and ompK36), which reduces outer membrane permeability to carbapenems [66]. The complexity and heterogeneity of ARGs among *K. pneumoniae* isolates emphasize the urgent demand for enhanced surveillance and effective intervention measures.

This study has several limitations. First, our use of short-read Illumina sequencing precludes complete assembly of plasmid structures and thus limits our ability to track horizontal gene transfer. Second, owing to the retrospective study design, we were unable to retrieve clinical outcome data (e.g., mortality) for individual patients, which prevented us from directly correlating genotypic features with clinical severity. Third, single-hospital sampling introduces bias toward hospitalized patients and may fail to capture circulating community strains. Fourth, although we followed standardized protocols, the string test remains subject to variability due to inter-operator differences. Fifth, we acknowledge the absence of specific *K. pneumoniae* reference strains (hvKP and cKP) as controls in our hypervirulence assays. While *E. coli* ATCC 25922 was used for susceptibility testing, it is not suitable for validating hypermucoviscosity or virulence gene detection.

## 5. Conclusions

We retrospectively characterized 205 clinical *K. pneumoniae* isolates from Huzhou during 2020–2025. Hypervirulent strains were highly prevalent, with ST23-K1 and ST86-K2 as the dominant clones. The isolates exhibited high resistance to ampicillin, tetracycline and trimethoprim-sulfamethoxazole, while remaining largely susceptible to ceftazidime/avibactam, colistin and tigecycline. Given their retained antimicrobial activity, we recommend that the latter three agents be prioritized in empirical and targeted treatment algorithms for severe hvKP infections within Huzhou. We identified 11 *bla*_KPC-2_-positive isolates and one *mcr-1*-positive isolate, with diverse plasmids and high genomic diversity. Our findings demonstrate the co-occurrence of hypervirulence and antimicrobial resistance in local *K. pneumoniae*, highlighting the necessity of sustained molecular surveillance and optimized antibiotic use to prevent the spread of high-risk clones.

## Figures and Tables

**Table 1:** Antimicrobial susceptibility profile of 205 clinical *K. pneumoniae* strains.

Antibiotics	Range (µg/mL)	MIC50 (µg/mL)	MIC90 (µg/mL)	Breakpoint Interpretive Criteria (µg/mL)			Number (%)		
				S	I	R	S	I	R
CHL	4–32	8	>32	≤8	16	≥32	149 (72.68%)	14 (6.83%)	42 (20.49%)
SXT	0.5–8	≤0.5	>8	≤2/38	–	≥4/76	152 (74.15%)	0 (0%)	53 (25.85%)
CT	0.25–8	≤0.25	2	≤2	–	>2	199 (97.07%)	0 (0%)	6 (2.93%)
ETP	0.25–8	≤0.25	>8	≤0.5	1	≥2	177 (86.34%)	0 (0%)	28 (13.66%)
MEM	0.125–2	≤0.12	>2	≤1	2	≥4	179 (87.32%)	2 (0.97%)	24 (11.71%)
CTX	0.25–16	≤0.25	>16	≤1	2	≥4	157 (76.59%)	0 (0%)	48 (23.41%)
CAZ	0.25–16	0.5	>16	≤4	8	≥16	174 (84.88%)	3 (1.46%)	28 (13.66%)
CZA	0.25–8	≤0.25	2	≤8/4	–	≥16/4	202 (98.54%)	0 (0%)	3 (1.46%)
TET	1–16	2	>16	≤4	8	≥16	147 (71.71%)	2 (0.97%)	56 (27.32%)
TIG	0.25–8	≤0.25	2	≤0.5	–	>0.5	188 (91.71%)	0 (0%)	17 (8.29%)
CIP	0.015–2	0.06	>2	≤0.25	0.5	≥1	150 (73.17%)	16 (7.81%)	39 (19.02%)
NAL	4–32	≤4	>32	≤16	–	≥32	168 (81.95%)	0 (0%)	37 (18.05%)
AZM	2–64	8	64	≤16	–	≥32	164 (80.00%)	0 (0%)	41 (20.00%)
AMI	4–64	≤4	>64	≤4	8	≥16	184 (89.76%)	0 (0%)	21 (10.24%)
STR	4–32	≤4	>32	≤8	16	≥32	160 (78.05%)	10 (4.88%)	35 (17.07%)
AMP	2–32	32	>32	≤8	16	≥32	6 (2.93%)	48 (23.41%)	151 (73.66%)
AMS	2–32	4	>32	≤8/4	16/8	≥32/16	150 (73.17%)	9 (4.39%)	46 (22.44%)

## Data Availability

The data presented in this study are openly available in GenBank. The Bioproject number PRJNA1081623 and PRJNA1423361. The original contributions presented in this study are included in the article/Supplementary Material. Further inquiries can be directed to the corresponding author.

## Supplementary Materials

The following supporting information can be downloaded at https://www.mdpi.com/article/10.3390/microorganisms14071498/s1: Table S1. Overview of the genome sequences; Table S2. Antimicrobial susceptibility profile of *bla*_KPC-2_-positive and *mcr-1*-positive *K. pneumoniae*; Table S3. Virulence factors of 205 *K. pneumoniae* isolates; Table S4. A comprehensive summary of nine “superbugs” (*bla*_KPC-2_ and hvKP); Table S5. ARGs of 205 *K. pneumoniae* isolates; Table S6. SNP variation loci of *mcr-1*-harboring *K. pneumoniae* isolates.

## References

[1] Choby J.E., Howard-Anderson J., Weiss D.S. (2020). Hypervirulent *Klebsiella pneumoniae*–clinical and molecular perspectives. J. Intern. Med..

[2] Wyres K.L., Lam M.M.C., Holt K.E. (2020). Population genomics of *Klebsiella pneumoniae*. Nat. Rev. Microbiol..

[3] Russo T.A., Marr C.M. (2019). Hypervirulent *Klebsiella pneumoniae*. Clin. Microbiol. Rev..

[4] Lin X.C., Li C.L., Zhang S.Y., Yang X.F., Jiang M. (2023). The Global and Regional Prevalence of Hospital-Acquired Carbapenem-Resistant *Klebsiella pneumoniae* Infection: A Systematic Review and Meta-analysis. Open Forum Infect. Dis..

[5] Heng H., Yang X., Ye L., Tang Y., Guo Z., Li J., Chan E.W., Zhang R., Chen S. (2024). Global genomic profiling of *Klebsiella pneumoniae*: A spatio-temporal population structure analysis. Int. J. Antimicrob. Agents.

[6] Mohammed T.M., Ayaid K.Z. (2025). Multidrug-resistant *Klebsiella pneumoniae* isolated from an infected wound in Iraq. World J. Exp. Biosci..

[7] Yang X., Sun Q., Li J., Jiang Y., Li Y., Lin J., Chen K., Chan E.W., Zhang R., Chen S. (2022). Molecular epidemiology of carbapenem-resistant hypervirulent *Klebsiella pneumoniae* in China. Emerg. Microbes Infect..

[8] Liu Y.C., Cheng D.L., Lin C.L. (1986). *Klebsiella pneumoniae* liver abscess associated with septic endophthalmitis. Arch. Intern. Med..

[9] Pu D., Zhao J., Chang K., Zhuo X., Cao B. (2023). “Superbugs” with hypervirulence and carbapenem resistance in *Klebsiella pneumoniae*: The rise of such emerging nosocomial pathogens in China. Sci. Bull..

[10] Zhang R., Liu L., Zhou H., Chan E.W., Li J., Fang Y., Li Y., Liao K., Chen S. (2017). Nationwide Surveillance of Clinical Carbapenem-resistant Enterobacteriaceae (CRE) Strains in China. eBioMedicine.

[11] Liu X., Wu Y., Zhu Y., Jia P., Li X., Jia X., Yu W., Cui Y., Yang R., Xia W. (2022). Emergence of colistin-resistant hypervirulent *Klebsiella pneumoniae* (CoR-HvKp) in China. Emerg. Microbes Infect..

[12] Mobasseri G., Teh C.S.J., Ooi P.T., Thong K.L. (2019). The emergence of colistin-resistant *Klebsiella pneumoniae* strains from swine in Malaysia. J. Glob. Antimicrob. Resist..

[13] Zheng Z., Liu L., Ye L., Xu Y., Chen S. (2023). Genomic insight into the distribution and genetic environment of blaIMP-4 in clinical carbapenem-resistant *Klebsiella pneumoniae* strains in China. Microbiol. Res..

[14] Liao W., Liu Y., Zhang W. (2020). Virulence evolution, molecular mechanisms of resistance and prevalence of ST11 carbapenem-resistant *Klebsiella pneumoniae* in China: A review over the last 10 years. J. Glob. Antimicrob. Resist..

[15] Wang M., Earley M., Chen L., Hanson B.M., Yu Y., Liu Z., Salcedo S., Cober E., Li L., Kanj S.S. (2022). Clinical outcomes and bacterial characteristics of carbapenem-resistant *Klebsiella pneumoniae* complex among patients from different global regions (CRACKLE-2): A prospective, multicentre, cohort study. Lancet Infect. Dis..

[16] Hu F., Pan Y., Li H., Han R., Liu X., Ma R., Wu Y., Lun H., Qin X., Li J. (2024). Carbapenem-resistant *Klebsiella pneumoniae* capsular types, antibiotic resistance and virulence factors in China: A longitudinal, multi-centre study. Nat. Microbiol..

[17] CLSI (2024). Performancestandards for Antimicrobial Susceptibility Testing.

[18] The European and Committee on Antimicrobial Susceptibility Testing (2023). Breakpoint Tables or Interpretation of MICs and Zone Diameters. Version 13.0. (2023-01-01).

[19] Kumabe A., Kenzaka T. (2014). String test of hypervirulent *Klebsiella pneumonia*. QJM Int. J. Med..

[20] Bolger A.M., Lohse M., Usadel B. (2014). Trimmomatic: A flexible trimmer for Illumina sequence data. Bioinformatics.

[21] Bankevich A., Nurk S., Antipov D., Gurevich A.A., Dvorkin M., Kulikov A.S., Lesin V.M., Nikolenko S.I., Pham S., Prjibelski A.D. (2012). SPAdes: A new genome assembly algorithm and its applications to single-cell sequencing. J. Comput. Biol..

[22] Lam M.M.C., Wick R.R., Watts S.C., Cerdeira L.T., Wyres K.L., Holt K.E. (2021). A genomic surveillance framework and genotyping tool for *Klebsiella pneumoniae* and its related species complex. Nat. Commun..

[23] Alcock B.P., Huynh W., Chalil R., Smith K.W., Raphenya A.R., Wlodarski M.A., Edalatmand A., Petkau A., Syed S.A., Tsang K.K. (2023). CARD 2023: Expanded curation, support for machine learning, and resistome prediction at the Comprehensive Antibiotic Resistance Database. Nucleic Acids Res..

[24] Buchfink B., Reuter K., Drost H.G. (2021). Sensitive protein alignments at tree-of-life scale using DIAMOND. Nat. Methods.

[25] Croucher N.J., Page A.J., Connor T.R., Delaney A.J., Keane J.A., Bentley S.D., Parkhill J., Harris S.R. (2015). Rapid phylogenetic analysis of large samples of recombinant bacterial whole genome sequences using Gubbins. Nucleic Acids Res..

[26] Minh B.Q., Schmidt H.A., Chernomor O., Schrempf D., Woodhams M.D., von Haeseler A., Lanfear R. (2020). IQ-TREE 2: New Models and Efficient Methods for Phylogenetic Inference in the Genomic Era. Mol. Biol. Evol..

[27] Xie J., Chen Y., Cai G., Cai R., Hu Z., Wang H. (2023). Tree Visualization by One Table (tvBOT): A web application for visualizing, modifying and annotating phylogenetic trees. Nucleic Acids Res..

[28] Chen Y., Chen Y., Liu P., Guo P., Wu Z., Peng Y., Deng J., Kong Y., Cui Y., Liao K. (2022). Risk factors and mortality for elderly patients with bloodstream infection of carbapenem resistance *Klebsiella pneumoniae*: A 10-year longitudinal study. BMC Geriatr..

[29] Li J., Ren J., Wang W., Wang G., Gu G., Wu X., Wang Y., Huang M., Li J. (2018). Risk factors and clinical outcomes of hypervirulent *Klebsiella pneumoniae* induced bloodstream infections. Eur. J. Clin. Microbiol. Infect. Dis..

[30] Lei T.Y., Liao B.B., Yang L.R., Wang Y., Chen X.B. (2024). Hypervirulent and carbapenem-resistant *Klebsiella pneumoniae*: A global public health threat. Microbiol. Res..

[31] Arias C.A., Murray B.E. (2009). Antibiotic-resistant bugs in the 21st century—A clinical super-challenge. N. Engl. J. Med..

[32] Ogunlana L., Kaur D., Shaw L.P., Jangir P., Walsh T., Uphoff S., MacLean R.C. (2023). Regulatory fine-tuning of mcr-1 increases bacterial fitness and stabilises antibiotic resistance in agricultural settings. ISME J..

[33] Ding L., Shen S., Chen J., Tian Z., Shi Q., Han R., Guo Y., Hu F. (2023). *Klebsiella pneumoniae* carbapenemase variants: The new threat to global public health. Clin. Microbiol. Rev..

[34] Yan W., Xu D., Shen Y., Dong F., Ji L. (2024). Molecular epidemiology of string test-positive *Klebsiella pneumoniae* isolates in Huzhou, China, 2020–2023. Front. Cell. Infect. Microbiol..

[35] Biswas S., Brunel J.M., Dubus J.C., Reynaud-Gaubert M., Rolain J.M. (2012). Colistin: An update on the antibiotic of the 21st century. Expert Rev. Anti-Infect. Ther..

[36] Jia M., Zhang J., Feng J., Zhuang Y., Xu Z., Yuan L., Luo J., Hong L., Xia J., Wu H. (2025). Epidemiological and genomic insights of mcr-1-positive colistin-resistant *Klebsiella pneumoniae* species complex strains from wastewater treatment plants in Shanghai. Environ. Pollut..

[37] Zhuang H.H., Qu Q., Long W.M., Hu Q., Wu X.L., Chen Y., Wan Q., Xu T.T., Luo Y., Yuan H.Y. (2025). Ceftazidime/avibactam versus polymyxin B in carbapenem-resistant *Klebsiella pneumoniae* infections: A propensity score-matched multicenter real-world study. Infection.

[38] Maguire M., DeLappe N., Clarke C., Touhy A., Carlino-MacDonald U., Hutson A., Cormican M., Brennan W., Devane G., Morris D. (2025). Genomic and phylogenetic analysis of hypervirulent *Klebsiella pneumoniae* ST23 in Ireland. Microb. Genom..

[39] Navon-Venezia S., Kondratyeva K., Carattoli A. (2017). *Klebsiella pneumoniae*: A major worldwide source and shuttle for antibiotic resistance. FEMS Microbiol. Rev..

[40] Wyres K.L., Wick R.R., Gorrie C., Jenney A., Follador R., Thomson N.R., Holt K.E. (2016). Identification of Klebsiella capsule synthesis loci from whole genome data. Microb. Genom..

[41] Podschun R., Ullmann U. (1998). *Klebsiella* spp. as nosocomial pathogens: Epidemiology, taxonomy, typing methods, and pathogenicity factors. Clin. Microbiol. Rev..

[42] Wyres K.L., Wick R.R., Judd L.M., Froumine R., Tokolyi A., Gorrie C.L., Lam M.M.C., Duchêne S., Jenney A., Holt K.E. (2019). Distinct evolutionary dynamics of horizontal gene transfer in drug resistant and virulent clones of *Klebsiella pneumoniae*. PLoS Genet..

[43] Anantharajah A., Deltombe M., de Barsy M., Evrard S., Denis O., Bogaerts P., Hallin M., Miendje Deyi V.Y., Pierard D., Bruynseels P. (2022). Characterization of hypervirulent *Klebsiella pneumoniae* isolates in Belgium. Eur. J. Clin. Microbiol. Infect. Dis..

[44] Lee I.R., Molton J.S., Wyres K.L., Gorrie C., Wong J., Hoh C.H., Teo J., Kalimuddin S., Lye D.C., Archuleta S. (2016). Differential host susceptibility and bacterial virulence factors driving Klebsiella liver abscess in an ethnically diverse population. Sci. Rep..

[45] Russo T.A., Olson R., Fang C.T., Stoesser N., Miller M., MacDonald U., Hutson A., Barker J.H., La Hoz R.M., Johnson J.R. (2018). Identification of Biomarkers for Differentiation of Hypervirulent *Klebsiella pneumoniae* from Classical *K. pneumoniae*. J. Clin. Microbiol..

[46] David S., Reuter S., Harris S.R., Glasner C., Feltwell T., Argimon S., Abudahab K., Goater R., Giani T., Errico G. (2019). Epidemic of carbapenem-resistant *Klebsiella pneumoniae* in Europe is driven by nosocomial spread. Nat. Microbiol..

[47] Shi Q., Lan P., Huang D., Hua X., Jiang Y., Zhou J., Yu Y. (2018). Diversity of virulence level phenotype of hypervirulent *Klebsiella pneumoniae* from different sequence type lineage. BMC Microbiol..

[48] Wyres K.L., Nguyen T.N.T., Lam M.M.C., Judd L.M., van Vinh Chau N., Dance D.A.B., Ip M., Karkey A., Ling C.L., Miliya T. (2020). Genomic surveillance for hypervirulence and multi-drug resistance in invasive *Klebsiella pneumoniae* from South and Southeast Asia. Genome. Med..

[49] Wu Y., Pu F., Yan Z., Zhang Y., Chen K., Li S., Wang Y., Lun H., Qu T., Wang J. (2025). Geographic containment and virulence-resistance trade-offs drive the evolution of hypervirulent *Klebsiella pneumoniae*. iMeta.

[50] Shon A.S., Bajwa R.P., Russo T.A. (2013). Hypervirulent (hypermucoviscous) *Klebsiella pneumoniae*: A new and dangerous breed. Virulence.

[51] Catalán-Nájera J.C., Garza-Ramos U., Barrios-Camacho H. (2017). Hypervirulence and hypermucoviscosity: Two different but complementary *Klebsiella* spp. phenotypes?. Virulence.

[52] Struve C., Bojer M., Krogfelt K.A. (2009). Identification of a conserved chromosomal region encoding *Klebsiella pneumoniae* type 1 and type 3 fimbriae and assessment of the role of fimbriae in pathogenicity. Infect. Immun..

[53] Padilla E., Llobet E., Doménech-Sánchez A., Martínez-Martínez L., Bengoechea J.A., Albertí S. (2010). *Klebsiella pneumoniae* AcrAB efflux pump contributes to antimicrobial resistance and virulence. Antimicrob. Agents Chemother..

[54] Li L., Gao X., Li M., Liu Y., Ma J., Wang X., Yu Z., Cheng W., Zhang W., Sun H. (2024). Relationship between biofilm formation and antibiotic resistance of *Klebsiella pneumoniae* and updates on antibiofilm therapeutic strategies. Front. Cell. Infect. Microbiol..

[55] Barkat A.K., Muhammad A.U.D., Mohammed T.M., Maimoona B., Habib U. (2025). Antagonism and Antibiofilm Activity of Sterile Microbiota Growth Medium against *Klebsiella pneumoniae* In Vitro. World J. Exp. Biosci..

[56] Zhu C., Li C., Lai C.K.C., Ng R., Chau K.Y., Wong K.T., Lo N.W.S., Barua N., Yang Y., Liyanapathirana V. (2021). Longitudinal Genomic Characterization of Carbapenemase-producing Enterobacteriaceae (CPE) Reveals Changing Pattern of CPE Isolated in Hong Kong Hospitals. Int. J. Antimicrob. Agents.

[57] Tian D., Liu X., Chen W., Zhou Y., Hu D., Wang W., Wu J., Mu Q., Jiang X. (2022). Prevalence of hypervirulent and carbapenem-resistant *Klebsiella pneumoniae* under divergent evolutionary patterns. Emerg. Microbes Infect..

[58] Huang Y.H., Chou S.H., Liang S.W., Ni C.E., Lin Y.T., Huang Y.W., Yang T.C. (2018). Emergence of an XDR and carbapenemase-producing hypervirulent *Klebsiella pneumoniae* strain in Taiwan. J. Antimicrob. Chemother..

[59] Li L., Liang J., Zhang H., Guo J., Li S., Li M. (2025). Emergence and clinical challenges of ST11-K64 carbapenem-resistant *Klebsiella pneumoniae*: Molecular insights and implications for antimicrobial resistance and virulence in Southwest China. BMC Infect. Dis..

[60] Lian J., Li Q., Peng C., Lin T., Du H., Tang C., Zhang X. (2025). Molecular and epidemiological characterization of carbapenem-resistant hypervirulent *Klebsiella pneumoniae* in Huaian, China (2022–2024): A retrospective study. Front. Cell. Infect. Microbiol..

[61] Cassini A., Högberg L.D., Plachouras D., Quattrocchi A., Hoxha A., Simonsen G.S., Colomb-Cotinat M., Kretzschmar M.E., Devleesschauwer B., Cecchini M. (2019). Attributable deaths and disability-adjusted life-years caused by infections with antibiotic-resistant bacteria in the EU and the European Economic Area in 2015: A population-level modelling analysis. Lancet Infect. Dis..

[62] Righi E., Mutters N.T., Guirao X., Del Toro M.D., Eckmann C., Friedrich A.W., Giannella M., Kluytmans J., Presterl E., Christaki E. (2023). ESCMID/EUCIC clinical practice guidelines on perioperative antibiotic prophylaxis in patients colonized by multidrug-resistant Gram-negative bacteria before surgery. Clin. Microbiol. Infect..

[63] El-Sayed Ahmed M.A.E., Zhong L.L., Shen C., Yang Y., Doi Y., Tian G.B. (2020). Colistin and its role in the Era of antibiotic resistance: An extended review (2000–2019). Emerg. Microbes Infect..

[64] Liu Y.Y., Wang Y., Walsh T.R., Yi L.X., Zhang R., Spencer J., Doi Y., Tian G., Dong B., Huang X. (2016). Emergence of plasmid-mediated colistin resistance mechanism MCR-1 in animals and human beings in China: A microbiological and molecular biological study. Lancet Infect. Dis..

[65] Wang X., Yu D., Chen L. (2023). Antimicrobial resistance and mechanisms of epigenetic regulation. Front. Cell. Infect. Microbiol..

[66] Rosas N.C., Wilksch J., Barber J., Li J., Wang Y., Sun Z., Rocker A., Webb C.T., Perlaza-Jiménez L., Stubenrauch C.J. (2023). The evolutionary mechanism of non-carbapenemase carbapenem-resistant phenotypes in *Klebsiella* spp.. eLife.

